# Three Cases of Fractured Scull

**Published:** 1800-01

**Authors:** 

**Affiliations:** Wisbeach


### *Three Cafes of Fractured Scull:—Communicated by Dr.* Skrimshire, *of Wifbeach.*

Gentlemen, *November* 21, 1799.

I SEND you the three following cafes for infertion in your Medical and Phyfical Journal, as ferving to illuftrate, and in fome degree fupport, the opinion of Mr. Abernethy, that the trepan is not fo often neceffary, as all the ancient, and many of the modern furgeons, have inculcated.

In drawing up the firft cafe, I have deviated from the ufual mode of giving daily reports, and have detailed the fymptoms and their changes, the practice and its effects, in one continued hiftory. This I have done, becaufe as either I myfelf, or Dr. Fawffett, was with this patient both day and night, every new fymptom was obferved as foon as it arofe, and the proper treatment immediately had recourfe to, fo that it would be impoffible to divide it into regular reports. The fame caufe has made the defcription more minute than otherwife it would have been; but as the accuracy of it may be depended upon, and the cafe is in many refpects a remarkable one, I truft it will not be thought tedious.

---

On July 17th, 1799, Mrs. M. a lady aged 26, was thrown with violence from an open carriage upon a ftone pavement. She was taken up fenfelefs, conveyed to the neareft houfe, and put under the care of a furgeon, who was on the fpot at the time of the accident. In a few minutes fhe began to recover; fome ftrong brandy and water was given, and her fenfes were foon reftored. Her nofe bled confiderably, her face was much bruifed under both eyes and acrofs the nofe, and a fmall fwelling was was perceived on the right ſide of the head. About a quarter of an hour after the accident, ſhe became ſick, and felt a peculiar tingling ſenſation in her extremities, to which ſucceeded vomiting; this ſame ſenſation recurred three different times, and was each time ſucceeded by vomiting. It being late in the evening, ſhe now went to bed, and paſſed a pretty good night; in the morning ſhe complained of being much bruiſed, but ſeemed otherwiſe well. At nine o'clock, after drinking ſome warm tea, ſhe became ſick, had convulſive twitchings of the ſmall muſcles about the noſe and mouth, and had alſo the ſame ſenſation in her extremities as yeſterday, but more conſtant, and, except at the commencement, not attended with vomiting. The muſcles of the hands too were ſpaſmodically contracted, ſo that her fingers were drawn together, but the hand not cloſed. The ſurgeon preſcribed ether to be taken freely in brandy and water, and ordered her arms to be rubbed with ſpirits. The ſymptoms rather increaſed than abated during the whole morning; and to thoſe above deſcribed, were ſoon added thoſe of involuntary laughing and ſinging. She alſo ſpoke in a ſharp, quick manner, but not incoherently. Her anſwers were pertinent, but her whole converſation rapid. Theſe ſymptoms being conſidered hyſterical, the ether was continued. It was in this ſtate that I firſt ſaw her, about two o'clock in the afternoon, on the day after the accident.

Apprehending ſomething more ſerious than hyſterics, I immediately examined her head, but only found a ſmall ſwelling, without any evident appearance of further injury than a bruiſe. She complained of ſome uneaſineſs in her bowels, and her left groin was very conſiderably bruiſed. Her pulſe was quick, but regular and equal. She ſnorted once very loudly whilſt I was preſent, and I was informed had done ſo twice before; her breathing was otherwiſe perfectly natural.

Having ordered them to give no more ether until I returned, I went immediately to meet the ſurgeon: as he thought venæſection might aggravate the hyſterical ſymptoms, I propoſed waiting for the lady's father, who was a medical man, and was expected every minute. When he arrived, venæſection was thought proper, and immediately had recourſe to. The bad ſymptoms were relieved by it, and ſhe lay much more compoſed. A draught with half an ounce of ol. ricini was ordered to be taken directly. The oil made her ſick, and was partly thrown up. About an hour after the bleeding, the ſymptoms returned; her eyes were protruded, and the pupils dilated. I again examined the ſwelling of the head, ſtrongly ſuſpecting this to be the ſeat of the miſchief. The tumour appeared as before, and being circumſcribed, gave the ſenſation to the touch, as if the bone bone was depressed, but this proved afterwards to be fallacious. I was, however, convinced that this was the seat of the injury, when I perceived that her eyes were evidently more distorted whilst one of the medical gentlemen was pressing on the tumour. Thinking an operation indispensible, I called in my very worthy friend, Dr. Fawssett, who was then accidentally in the town. His opinion of the case exactly coincided with mine; and after consulting with the surgeons, it was agreed to make an incision across the tumour, to examine the state of the cranium, and thereby to be regulated, as to the necessity of any farther operation. A longitudinal incision was therefore made from near the middle of the parietal bone, in a straight direction to the coronal suture. A small quantity of uncoagulated blood escaped from the tumuor, whether from under the pericranium or not, could not be ascertained, as this was divided by the same incision with the teguments; the pericranium, however, had a very healthy appearance. It was now fully evident, that the parietal bone was fractured, a fissure of two or three inches in extent being easily observable, nearly in the direction of the surgeon's incision. The bone was fortunately in no part of it depressed, and as our patient felt immediate relief, there was no indication for applying the trepan. The wound was dressed lightly; the convulsive twitchings, as well as the tingling sensation in the extremities, were no longer felt.

As our patient had had but one, and that a very costive stool, a common enema was given in the evening, after which she took a draught, with T^ra^ Opii et Vini Antimonialis āā g^tt^ x. The whole night was passed in a very calm, composed state, and she slept some hours. On the morning of the 19th, between seven and eight o'clock, she had two dark-coloured stools, attended with very considerable uneasiness in her bowels. At eight the convulsive distortions of the face returned, but in a slight degree, and not attended with any of the other symptoms. I removed the dressings, and washed the wound of the head with warm water, and had ℥ viij of blood taken from the arm, after which the spasms did not return. A saline draught with a few drops of antimonial wine was given every four hours, to produce a gentle diaphoresis. The application of warm flannels relieved the uneasiness in the bowels. Her food was ordered, to be broths and ripe fruit. The room was darkened, and every noise avoided; she was placed with her head elevated, and desired to exert herself as little as possible in speaking or otherwise. The remainder of this day and the whole of the night passed over favourably; a draught with T^ra^ Opii et Vini Antimonialis āā g^tt^ v. taken at 10 P.M. and repeated at 2 A.M. procured several hours of refreshing sleep.

On the 20th, no return of unfavourable ſymptoms. The clyſter was repeated in the morning, and the two anodyne draughts at night, with the ſame good effects as before. Pulſe and ſkin natural.

On the 21ſt, ſhe was ſo well as to be removed to another room without any inconvenience. She exerted herſelf a good deal in converſation during the morning, but at noon her ſpirits flagged; ſhe became very low and faint, and began to be diſagreeably affected by noiſes. In a few hours ſhe complained of very violent head-ach, and a ſenſe of heat, chiefly in the forehead and temples, which felt as if tightly bound. Noiſes ſo ſlight as ſcarcely to be heard by thoſe in the ſame room, produced a jarring ſenſation in her head, that almoſt diſtracted her; the admiſſion of light too increaſed the pain. Slight but temporary relief was experienced from applying freſh dreſſings to the wound, as was the caſe from bathing the forehead and temples with a ſpirituous embrocation. The whole of the hair was now cut off, and her head kept uncovered; ℥ xij of blood were drawn from the arm. The pain ſoon became leſs acute, and the burning heat and diſtreſs from noiſe was leſs. The ſenſe of tightneſs with a dull head-ach remained, and ſhe was ſo very faint and low, as often to approach nearly to ſyncope. A ſpoonful of ſago with a very little wine was given every half hour. About 6 P.M. a bliſter was applied to the nape of the neck, and two leeches to each temple. At the time the leeches were preparing, a few drops of blood came from her noſe on blowing it, which gave very great relief to her head for a minute or two. The bleeding from the leeches likewiſe was evidently beneficial. About 10 P.M. our patient, thinking ſhe had experienced ſome relief from the bliſter, propoſed having another; Dr. Fawſſett therefore ordered another behind each ear, and two more leeches to the temples. The dull head-ach, together with the uneaſineſs from the bliſters, prevented her getting any ſleep, except now and then for a few minutes; but towards morning the head-ach abated. She had dyſuria during the night, and for an hour or two was unable to retain her urine. This, however, being the effect of the bliſters, was ſoon removed by drinking plentifully of gum diſſolved in milk and water. During this time, about 2 A.M. ſhe told me, that on paſſing a ſmall quantity of urine, ſhe had juſt experienced a very great and ſudden relief to her head; it was, however, but temporary.

On the 22d and 23d, Mrs. M. continued remarkably faint and low, and had a dull, uneaſy ſenſation in her head, though little pain, and leſs diſtreſs than before from noiſes. She took a little broth or ſago frequently, and now and then a little

ſherry

ſherry and water, and ate often of ripe fruit. The anodynes, as preſcribed on the 19th, were given each night, and the body kept open by clyſters.

On the 24th, 25th, and 26th, the head-ach gradually abated, ſo that Mrs. M. was now able to ſit up an hour or two.

On the 27th, ſhe ate ſome fowl for dinner, and had very little head-ach.

On 28th, in the evening, felt ſome head-ach, had more lowneſs than for ſome days paſt, and complained of heat about the wound, which was not more than half healed up. On 29th, leſs; and on 30th, ſcarcely any head-ach or heat about the wound. On 31ſt, ſhe was apparently quite well; and on 1ſt of Auguſt was conveyed home, a diſtance of 14 miles, without any ſenſe of fatigue. The next day about noon, however, ſhe began to complain of lowneſs, and dull head-ach, accompanied with flatulence and diſtention of the abdomen. Theſe ſymptoms were relieved by bitters and attention to diet; but on the 8th ſhe was conſiderably alarmed by an involuntary action of the left levator labii ſuperioris et alæ naſi, attended with a ſimilar ſenſation to that experienced before the operation. It was ſlight, and not perceived after a few minutes. She felt a prickling in the wound at the ſame time, from the appearance of which, and its diſpoſition to heal afterwards, I ſuſpect ſome trifling exfoliation took place, though the dreſſings were examined without finding any portion of bone. Since this, Mrs. M. has remained in as perfect health as before the accident, excepting that about a fortnight ago ſhe caught a cold, which was attended with a ſlight ſuppuration in the right ear, a complaint which ſhe never had before.

The abſence of coma in this caſe, and the relief experienced from a ſimple inciſion, make it doubtful whether there was any compreſſion of the brain, or whether the ſymptoms may not be attributed to tenſion, produced by the effuſion of blood under the integuments. Two inferences are, however, fairly deducible reſpecting the practice in ſuch caſes. Firſt, that attention ſhould be paid to the effects of inciſion before proceeding to the uſe of the trepan. Secondly, that caution is neceſſary in the uſe of ſtimulants; for, though there was in this caſe conſiderable concuſſion of the brain, which is ſaid by moſt practitioners to require the higheſt range of ſtimulants, yet the moſt alarming ſymptoms of inflammation afterwards occurred, notwithſtanding the early uſe of the lancet, and a ſtrict adherence to the antiphlogiſtic plan of treatment.

---

JULY 19, 1799.—I viſited W. D. a young gentleman aged four years, who had fallen the day before from a height of ten feet upon a brick pavement.

He vomited ſoon after he was taken up, and complained of a bruiſe on his head, but ſeemed otherwiſe quite well. The ſurgeon, who was immediately ſent for, eaſily diſcovered a very evident depreſſion of the right temporal bone, in the direction of its ſquamoſe ſuture, and likewiſe a fracture of the right parietal bone, extending from the middle of the ſquamoſe ſuture about one inch upwards. As no ſymptom of compreſſion was preſent, he merely applied a ſpirituous embrocation, and gave a gentle laxative, warning the child's friends of the bad ſymptoms that might very probably afterwards ariſe. I examined the head, and found the depreſſion and fracture as above deſcribed; but the former, as the ſurgeon informed me, was conſiderably leſs than it was yeſterday. No one bad ſymptom had come on, the boy was in good ſpirits, his pulſe and ſkin natural. As his phyſic had not operated, I ordered an enema; ℥ vi of blood were taken from the arm, and he was ordered a ſtrictly antiphlogiſtic regimen for two or three weeks.

This child has continued in full health and ſpirits. The depreſſed bone gradually roſe to its natural ſituation, ſo that in a few days it was not perceptible, and after a few weeks it was impoſſible to detect the ſituation of the fracture.

---

AUGUST 20th, 1799.

W. G. aged nine years, fell from the fore-horſe of a cart, upon a ſtone pavement, and the wheel immediately went over his head. I ſaw him about an hour after the accident, and found the whole of the left ſide of the head very much flattened; the temporal, and great part of the parietal bone, being ſo much depreſſed, that it would have taken twelve or fourteen folds of linen to have filled up the hollow to its natural ſhape. There was, beſides, a fracture of both bones, about one inch in extent, which croſſed the ſquamoſe ſuture.

The boy was comatoſe, but rouſed for a moment when ſpoken to. His breathing was laborious, pupils dilated, pulſe of natural velocity, but intermitting. He had vomited ſeveral times, had bled much from the noſe, and likewiſe from the right ear. In a conſultation with Mr. Clark, and Meſſrs. Skrimſhire, ſenior and junior, it was thought adviſeable to apply the trepan in two or more places, with a view to elevate the depreſſed bones. This was therefore propoſed to the parents of the boy, as apparently the only chance for his recovery. They, however, obſtinately refuſed, ſo that to put in practice the antiphlogiſtic plan, was all that was left us.

He

He was accordingly bled to ℥viij, an enema was adminiftered, and he was ordered to be kept quite ftill.

21ft. Morning. The depreffed portion of the bones is very evidently elevated, fo that the hollow is much lefs than it was laft night. He breathes more freely, but is ftill comatofe. When roufed, he complains of head-ach, putting his hand to his forehead; his fkin is hot, his pulfe quick and intermitting; has had no ftool from the clyfter.

℞. Natron tartaris. ℥ i. Mann. comm. Fruct. tamarind. aa ℥ ß. Inf. fennæ, ℥ v. f. M. Cujus capiat cochlearia ampla tria, 3tia. q. q. hora donec alvus foluta fiat.

Evening. The bones feem ftill more elevated. He has lefs of coma, but has been very reftlefs this afternoon. Skin hot, pulfe quick; has had no ftool yet.

Rep. Mift. aper. ad cochl. quatuor pro dos. Let him have plenty of ripe fruit.

22d. Morning. Has had a reftlefs night, is ftill comatofe, complains more of head-ach when roufed. Skin hot and dry, pulfe quick, but lefs frequently intermitting. The opening mixture has not operated. There is a fmall puffy, and circumfcribed tumor on the parietal bone, between the depreffion and crown of the head. There is no appearance of fracture or depreffion under the tumor, nor is it painful when preffed upon.

Inj. ftat. enema purg.

Evening. The clyfter procured a copious ftool. He has lefs coma, but much head-ach. Pulfe not fo quick, and lefs frequently intermitting.

Appl. tempori finiftro Hirud. N°. iv.

23d. Very little coma; complains of a dull, conftant head-ach; the tumor feems increafing; he has taken fome broth with appetite; has had no ftool fince that from the clyfter.

Rep. Enema.

24th. He has had a very good night, and is now free from both head-ach and coma. Pulfe irregular, but not intermitting. Belly open.

26th. He has continued quite free from complaint; has walked a little about his chamber. All his functions natural.

November 11th. Our patient has continued in perfect health. After a fortnight he refumed his ufual avocations.

The depreffion became gradually lefs for fome time, but that fide of the head is ftill very confiderably mifhapen. On or about the twelfth day after the accident, the tumor having been fome time ftationary, without any appearance of diminution, an incifion was made acrofs it. Its contents, being uncoagulated blood, were evacuated. The bone and pericranium were both found, and the wound healed in a few days.

The

The two last cases teach us, that in young subjects we may often expect even considerable depressions to be removed without any chirurgical aid. In such cases, therefore, we need be less anxious to perform an operation than in similar depressions in adults. The last case shews to what a great extent the brain may be injured without a fatal consequence; and the present mishapen appearance of the head is a proof, that the position and shape of the brain may be even permanently altered, without any bad consequence whatever, as the alteration in the form of the cranium is such, as must have induced an analogous one in that of its contents.

The happy termination of these three cases will, I trust, speak favourably of the strict antiphlogistic plan of treatment in injuries of the head.

